# Supplementary figures and images for: Dbf4 Zn-Finger Motif Is Specifically Required for Stimulation of Ctf19-Activated Origins in Saccharomyces cerevisiae
Source: Genes (Basel). 2022 Nov 24;13(12):2202. doi: 10.3390/genes13122202 (PMC9778208; doi:10.3390/genes13122202)

Figure S3

A

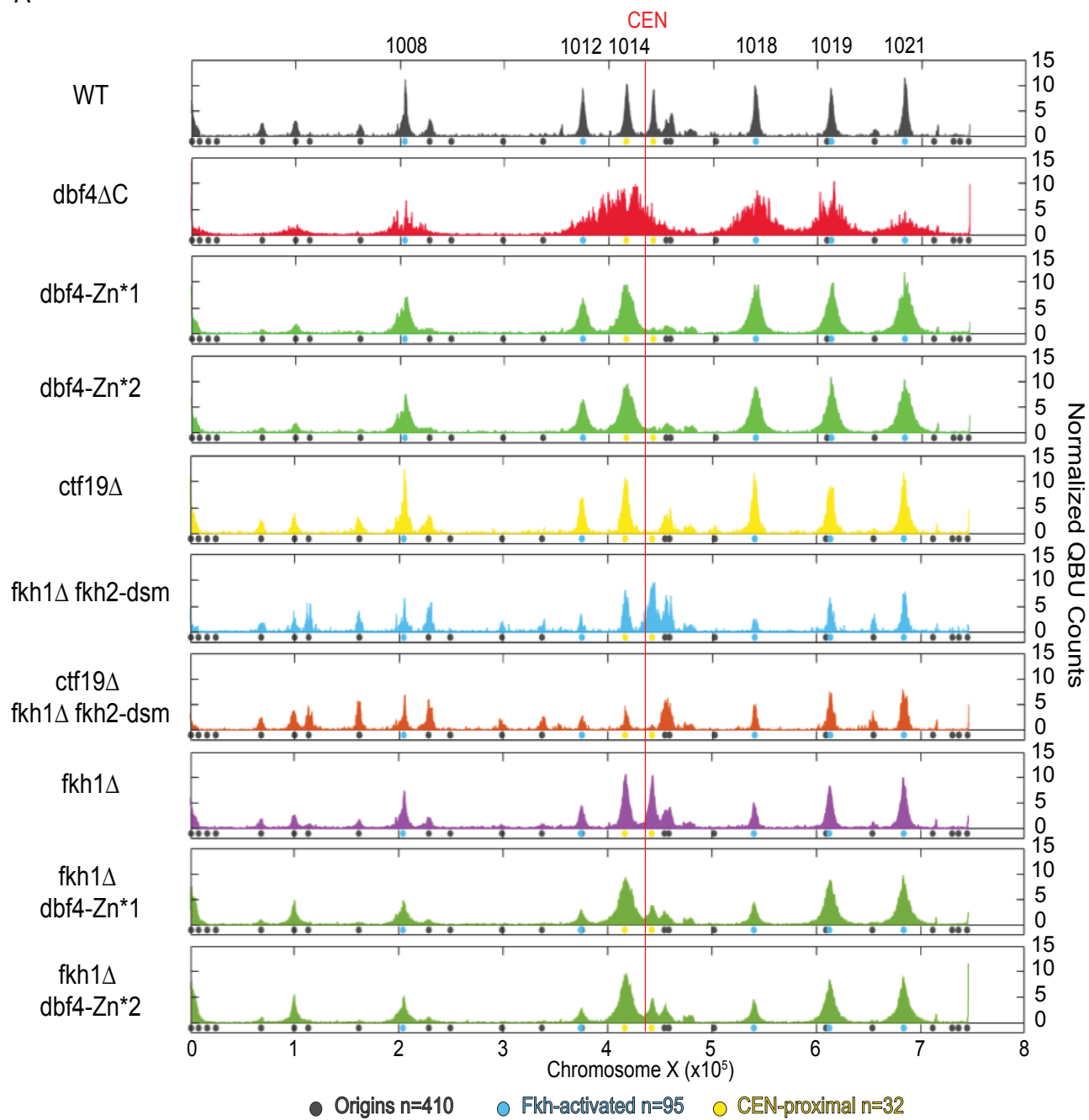

B

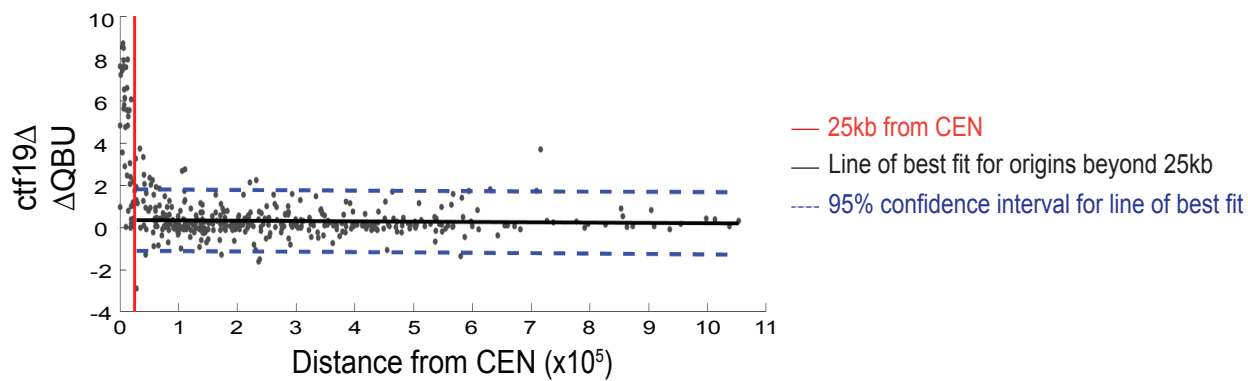

Supplement: Supplementary file 1 [file genes-13-02202-s001.zip › genes-1948321_supplementary_Updated/Dbf4_Zn_figS3_Resub_V2.pdf]

Figure S2

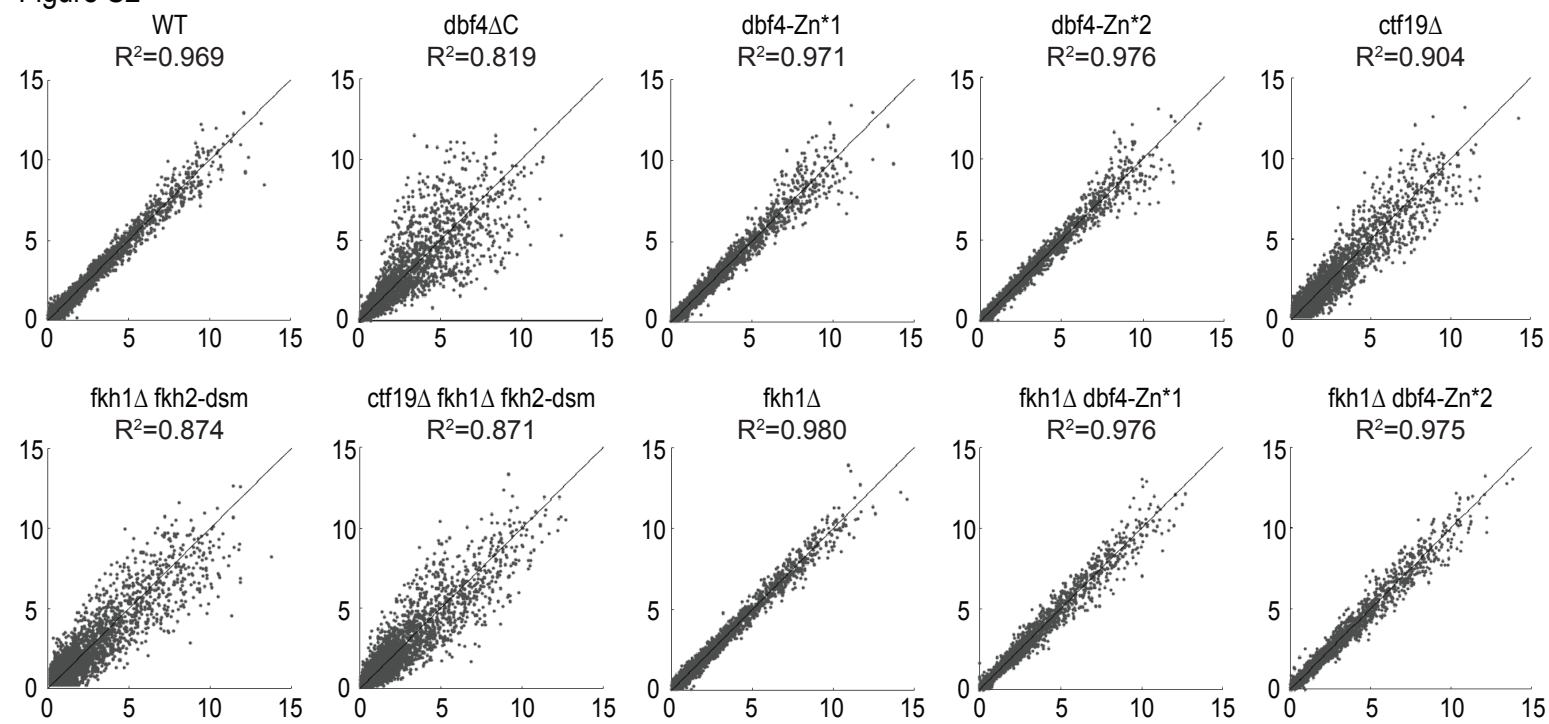

Supplement: Supplementary file 1 [file genes-13-02202-s001.zip › genes-1948321_supplementary_Updated/Dbf4_Zn_fig2S_ResubV1.pdf]

Figure S1

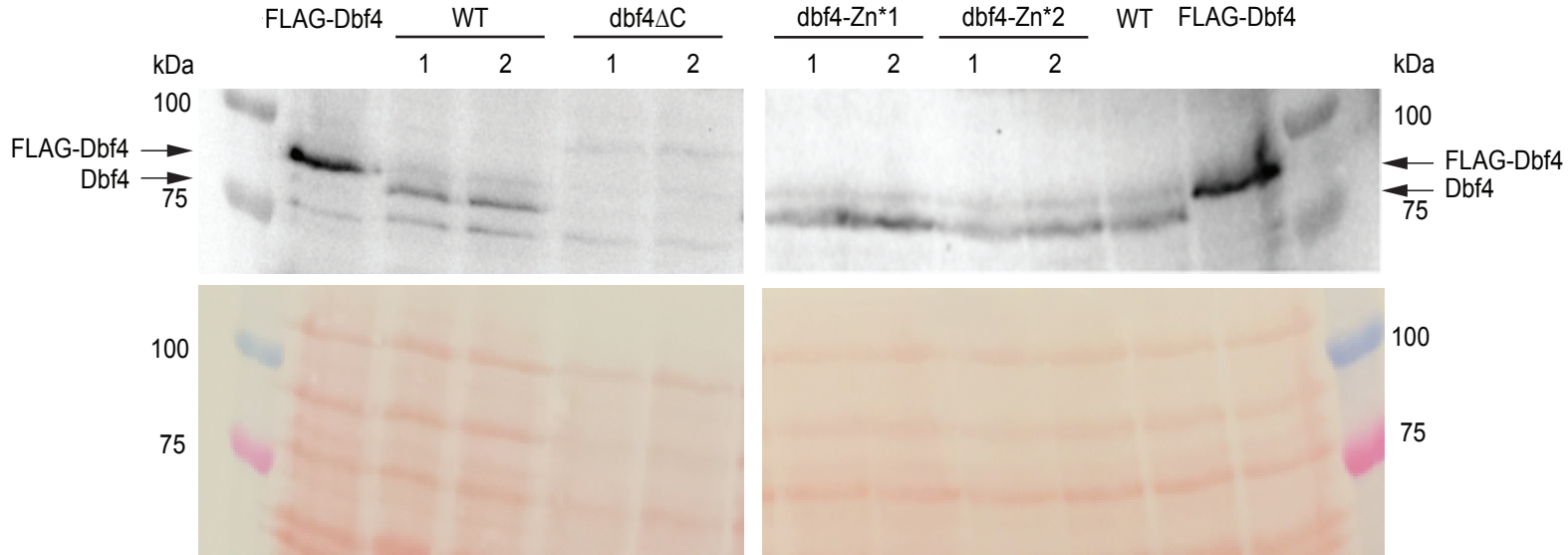

Supplement: Supplementary file 1 [file genes-13-02202-s001.zip › genes-1948321_supplementary_Updated/Dbf4_Zn_figS1_Resub_V1.pdf]
